# Fabrication of Multi-Layer Metal Oxides Structure for Colored Glass

**DOI:** 10.3390/ma14092437

**Published:** 2021-05-07

**Authors:** Akpeko Gasonoo, Hyeon-Sik Ahn, Eun-Jeong Jang, Min-Hoi Kim, Jin Seog Gwag, Jae-Hyun Lee, Yoonseuk Choi

**Affiliations:** 1 Research Institute of Printed Electronics & 3D Printing, Hanbat National University, Daejeon 34158, Korea; amoebatheo2009@gmail.com; 2 Department of Electronic Engineering, Hanbat National University, Daejeon 34158, Korea; princass123@naver.com (H.-S.A.); enjeong722@gmail.com (E.-J.J.); 3 Department of Creative Convergence Engineering, Hanbat National University, Daejeon 34158, Korea; mhkim8@hanbat.ac.kr; 4 Department of Physics, Yeungnam University, 214-1 Dae-dong, Gyeongsan 712-749, Korea; sweat3000@ynu.ac.kr

**Keywords:** BIPV, color glass, thin film interference, optical path-length difference, radio frequency sputtering, multi-layer film

## Abstract

This study proposes front colored glass for building integrated photovoltaic (BIPV) systems based on multi-layered derivatives of glass/MoO_3_/Al_2_O_3_ with a process technology developed to realize it. Molybdenum oxide (MoO_3_) and aluminum oxide (Al_2_O_3_) layers are selected as suitable candidates to achieve thin multi-layer color films, owing to the large difference in their refractive indices. We first investigated from a simulation based on wave optics that the glass/MoO_3_/Al_2_O_3_ multi-layer type offers more color design freedom and a cheaper fabrication process when compared to the glass/Al_2_O_3_/MoO_3_ multi-layer type. Based on the simulation, bright blue and green were primarily fabricated on glass. It is further demonstrated that brighter colors, such as yellow and pink, can be achieved secondarily with glass/MoO_3_/Al_2_O_3_/MoO_3_ due to enhanced multi-interfacial reflections. The fabricated color glasses showed the desired optical properties with a maximum transmittance exceeding 80%. This technology exhibits promising potential in commercial BIPV system applications.

## 1. Introduction

A novel approach to harvest solar energy from the envelope of buildings is to adopt building integrated photovoltaic (BIPV) systems that can efficiently generate renewable energy, mainly for the edifice [1,2]. This is necessary to ensure that a significant portion of the electricity generated is consumed within the built environment, therefore reducing the cost of electricity generation, transport, and distribution by smart grids [3]. Over the years, silicon-based solar cells such as single-crystalline silicon cells, multi-crystalline silicon cells, ribbon silicon, thin films, and amorphous cells have been investigated [4,5,6]. Recently, organic photovoltaics (OPVs), a technology that uses thin films of organic semiconductors to convert sunlight into electricity, have also been actively researched and have gained massive interest in recent years from the industrial sector [7,8,9]. OPVs enable cost-effective and eco-friendly roll-to-roll manufacturing in different sizes and shapes. They offer levels of energy consumption that are orders of magnitude smaller when compared to the manufacturing process for silicon-based solar cells. They also show better performance in low light; their electrolytes exhibit a better response to high temperatures, and they have a weak dependence on the angle of incidence [10,11,12]. These attributes are beneficial in integrating OPVs into building facades. The general adoption of BIPVs in various buildings paves the way for achieving efficient distributed energy generation. Even though BIPV systems have great potential to be applied in building envelopes, their ability to meet aesthetic considerations are unfortunately limited. In OPVs, different colors are obtained by employing different active materials exhibiting distinct absorption spectra [13]. Sometimes this color tuning approach can complicate fabrication processes for production and cause deviations in device performance among differently colored OPVs. Conventional silicon-based solar systems installed on large areas of buildings (roofs and facades) come in standard black or grey solar modules, mostly due to the antireflective coating layers within the PV cell [14]. This restricts aesthetic choice, which is not attractive to most users. One way to solve this problem is by adopting colored glasses that can hide the active components of the PV cell but transmit the non-reflected radiation entirely to the absorber with little or no absorption and can also exhibit stability over long-term use.

Some reports have investigated various colored glass concepts for BIPV systems. For instance, different pigment materials spin-coated on a gold surface for color tuning PV solar cells was reported by Guo et al. [15]. However, the color pigments absorb radiation and do not withstand degradation over time. The morpho butterfly effect, a bionic concept based on 3D photonic structures for making colored PV modules, has also been reported [16]. The proposed concept shows good angular stability, but it is complex and yields color glasses with low transmittance. Some recent technologies have investigated color films by the effect of interference from thin multi-layer films [17,18,19,20,21]. The color is derived based on the interference in the reflected light from the different interfaces of the thin multi-layer film [22,23,24]. A given incident light on a multi-layer film will experience reflection and transmission at each interface of the film. In most cases, the reflected rays interfere with each other, where their phase difference is determined by their optical path difference (OPD) [25,26]. Some inorganic layers have been used for the thin film optical applications, owing to their high transmittance and stable optical properties [21,27,28,29,30,31,32]. Unfortunately, most of the multi-layer structures that were adopted yielded very few achievable colors [19,20,21]. In other cases where low refractive index materials are used, thick layers are required to achieve bright, desirable colors. This approach is expensive, requires a long deposition time, yields layers with low transmittance, and cannot be applied in BIPV systems. A limited number of colored glasses achieved by depositing multi-layer films using atomic layer deposition have been reported by Kromatix™ technology [18,33]. Atomic layer deposition technique has been used in the nanotechnology industry for fabricating thin, uniform, and conformal films with accurate thickness control at less substrate damage [28]. However, atomic layer deposition may pose the challenge of slow growth rate when large-scale applications are required. Moreover, the effect of the structure on the optical properties of the multi-layer has been elusive and must be fully investigated. There is the need to achieve various colored glasses by depositing cheap thin layers through time-efficient deposition techniques such as physical vapor deposition. The critical understanding of the color film structure must also be comprehensively elucidated. Compared to other inorganic layers, metal oxides are cheaper and easier to synthesize and characterize [34]. This enables them to be potential materials to be adopted into applications that can be used anywhere in the environment. The large-scale development of non-oxide compounds has been limited due to the experimental challenge of synthesis and characterization, as non-oxide materials need to be prepared in atmospheres different from air/oxygen and may, in some cases, be air/moisture-sensitive, necessitating air-free environments and additional experimental skills.

In this paper, we investigated front colored glass for a BIPV system using multi-layer thin films composed of molybdenum oxide (MoO_3_) and aluminum oxide (Al_2_O_3_) layers. It was verified through computer simulation that the two metal oxide thin films with different refractive indices could be integrated to realize unique colored films with good transmittance. The large difference in the refractive index of MoO_3_ and Al_2_O_3_ offers a multi-layer that significantly changes the OPD of the reflected and transmitted spectra when the layer thickness is tuned. The MoO_3_ and Al_2_O_3_ layers were deposited via thermal evaporation and radio frequency reactive sputtering, respectively. Thermal vacuum evaporation enables the fabrication of multi-layer devices in which the thickness of each layer is controlled easily while sputtering deposition technique provides a simple apparatus, high deposition rate, and a low substrate temperature. Based on the simulation, bright and aesthetically considerable colors of blue, green, yellow, and pink color glasses were fabricated from the derivates of the glass/MoO_3_/Al_2_O_3_ multi-layer structure. A stable and uniformly colored glass with a high transmittance of over 80% is demonstrated. The multi-layer structure, composition, and fabrication allows a variety of colored films to be cheaply implemented in large, efficient BIPV systems. This investigation is crucial in fully understanding the mechanism of multi-layer thin colored films and their application in commercial BIPV systems and photonic devices.

## 2. Materials and Methods

**Figure 1:**
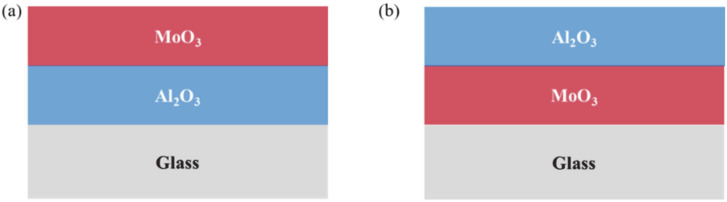
Schematic structure of (**a**) Type A and (**b**) Type B multi-layer.

Two different types of the multi-layer were investigated, as shown by the schematic structures in Figure 1. Types A and B were composed of glass/Al_2_O_3_/MoO_3_ and glass/MoO_3_/Al_2_O_3_, respectively. For the given incident light on the thin films, there were reflected rays produced at each boundary for which the OPDs depend on the thickness of the film layer, the refractive index of the film, and the angle of incidence.

High-purity MoO_3_ (≥99.5%) and 99.999% aluminum 100 mm in diameter were purchased from Sigma-Aldrich (Merck, Darmstadt, Germany) and iTASCO (Taewon Scientific Co., Ltd, Seoul, Korea), respectively. Argon (Ar) and oxygen (O_2_) gases were nominally 99.999% pure. All materials were used as received. Bare glass substrates (2.5 cm × 2.5 cm) with a thickness of 0.7 mm were used to fabricate the colored glasses after being sequentially cleaned with acetone and isopropyl alcohol in an ultrasonic bath, boiled in isopropyl alcohol, and dried in an oven at 150 °C for 15 min. The MoO_3_ layers were deposited by evaporation in a thermal evaporation chamber under 10^−7^ torr at deposition speed of 1 Å/s. The speed and thickness for the depositions were monitored using a quartz crystal monitor (STM-2XM, Sycon Instruments, Daejeon, Korea).

Al_2_O_3_ layers were deposited via a reactive sputtering system using a commercial aluminum target in a high-vacuum base pressure of 2.4 mTorr. Al_2_O_3_ depositions were carried out in plasma created from Ar and O_2_ gas mixtures by varying the partial pressures of gases with a mass flow controller (MFC) (TN2900, Nextron, Daejeon, Korea). The controlled plasma was formed adjacent to the substrate by a 300 W radio frequency energy, applied using a radio frequency power generator. The plasma dissociates the entering gases and processes them into ions and radicals that are deposited onto the substrate surface. A working pressure of 20 mTorr was maintained during sputtering. Pre-sputtering was conducted for about 5 min to stabilize the plasma state. The thickness of the Al_2_O_3_ layer deposited was 80 nm while the MoO_3_ layer varied between 60 nm and 100 nm depending on the colored glass.

The Essential Macleod software program by Thin Film Center Inc was used to simulate and analyze the optical characteristics of the structure of the color film. The optical characteristics of the deposited films were measured using ultraviolet visible near-infrared spectrophotometry (Lambda 950 UV-vis-NIR spectrophotometer, PerkinElmer, Waltham, MA, USA). The thicknesses of the films were confirmed from alpha step measurement while the refractive indices were measured using ellipsometry (Ellipso Technology, Elli-SE-U, SuWon-si, Korea).

## 3. Results and Discussion

### 3.1. Refractive Index and Reflectance Characteristics of MoO_3_ and Al_2_O_3_

**Figure 2:**
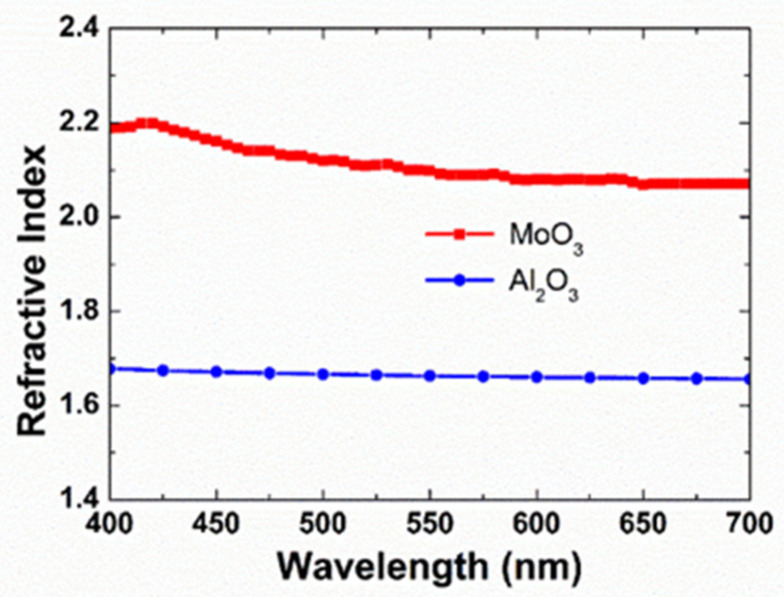
Measured refractive index of molybdenum oxide (MoO_3_) and aluminum oxide (Al_2_O_3_).

The measured refractive index of MoO_3_ and Al_2_O_3_ within the visible range is shown in Figure 2. The two layers exhibit large differences in refractive index, which are favorable for thin film optical applications. The refractive indices at 510 nm for glass, MoO_3_, and Al_2_O_3_ are 1.5, 2.1, and 1.7, respectively. Boudaoud et al. [30] have reported MoO_3_ thin films prepared on glass substrates by spray pyrolysis technique at a substrate temperature of 423 K. They reported an average value of the refractive index for MoO_3_ to be 2.1. We achieved a comparable refractive index for MoO_3_ films deposited on the substrate at room temperature using thermal evaporation. The refractive index of the Al_2_O_3_ film obtained by the reactive sputtering is in the range reported by other papers [31,32]. The simulation of the thin film interaction of the multi-layer types (Type A and Type B) are determined based on the measured refractive index of MoO_3_ and Al_2_O_3_.

### 3.2. Simulation and Analysis

**Figure 3:**
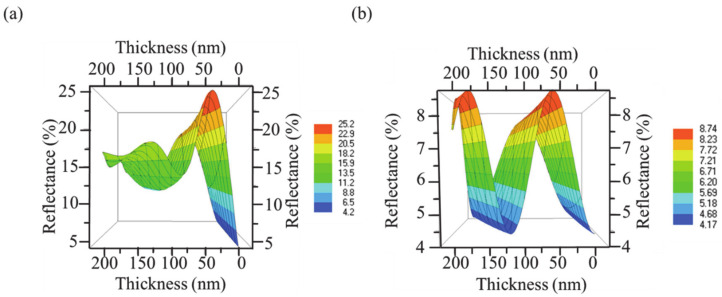
Simulation of the reflectance profiles of (**a**) MoO_3_ and (**b**) Al_2_O_3_.

The 3D simulation profile of the reflectance as a function of the thickness and wavelength of MoO_3_ and Al_2_O_3_ are shown in Figure 3, respectively. It is observed that, in the case of MoO_3_, an increase in thickness to 40 nm increased reflectance to 25%. Thereafter, it decreased to 13% at 100 nm, increased again to 18% at 140 nm, and decreased to 15% at 200 nm. Generally, the reflectance of Al_2_O_3_ is lower than MoO_3_ due to the low refractive index of the former. The reflectance of Al_2_O_3_ increased to 8.6% at 63 nm, decreased to 4.5% at 125 nm, increased back to 8.6% at 180 nm, and then decreased to 7.5% at 200 nm. The above characteristics demonstrate that the reflectance and transmittance characteristics of optical thin films are greatly affected by the refractive index and thickness of the thin film layers.

The effect of multi-layer structure on the thin film interference using computer simulations based on wave optics was further investigated. In the case of Type A, light traverses air, through to the MoO_3_ layer. Since the refractive index of MoO_3_ is higher than air, the reflection that occurs at the air–MoO_3_ boundary of the film introduces a 180° phase shift in the reflected wave [25,26]. The beam is then transmitted sequentially throughout this multi-layer type, where it encounters the next layer with a lower refractive index. There is no phase shift in the reflected light since the beam travels from a medium with a higher refractive index into a medium with a lower refractive index. However, in the multi-layer Type B, the beam is first transmitted from Al_2_O_3_ into MoO_3_, then from MoO_3_ into glass, and finally exits into air. Thus, the optical path traversed by the transmitted beam differs in the two multi-layer types. Generally, the color produced by the thin film interaction of each multi-layer depends on the constructive and destructive interferences of the reflected rays at each interface. The OPD of the reflected beams from the boundaries of each multi-layer type are obtained for each constructive and destructive interference of the reflected light [25,26]. The optical characteristics of the multi-layer types were first investigated in simulation. For brevity, a glass/Al_2_O_3_(40 nm)/MoO_3_ (80 nm) colored glass is referred to as “A40M80”, while glass/Al_2_O_3_(40 nm)/MoO_3_ (80 nm)/Al_2_O_3_(40 nm) is referred to as “A40M80A40”. Other colored glasses follow similar naming convention.

**Figure 4:**
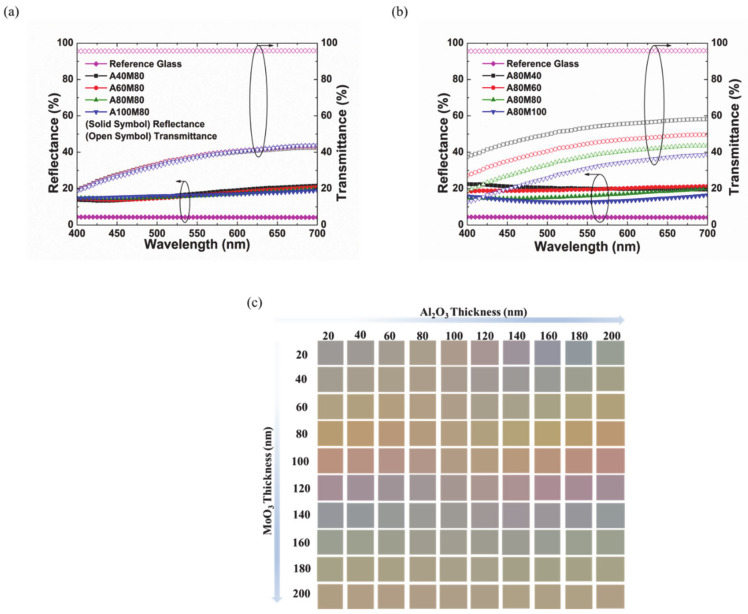
Reflectance and transmittance spectra of the Type A multi-layer (**a**) with 80 nm of MoO_3_ and 40 nm, 60 nm, 80 nm, and 100 nm of Al_2_O_3_ and (**b**) with 80 nm of Al_2_O_3_ and 40 nm, 60 nm, 80 nm, and 100 nm of MoO_3_. (**c**) Simulated color profile of the Type A multi-layer.

The reflectance and transmittance spectra of Type A for four multi-layers—with the same MoO_3_ layer thickness while that of the Al_2_O_3_ layer is varied from one multi-layer to the other—are shown in Figure 4a. The thickness of the MoO_3_ layer in each multi-layer is 80 nm while that of the Al_2_O_3_ layer is 40 nm, 60 nm, 80 nm, and 100 nm. It can be noticed that increasing the Al_2_O_3_ layer thickness decreases the reflectance marginally by 2% and increases the transmittance by same margin between 550 nm and 700 nm. Figure 4b shows the reflectance and transmittance spectra of Type A but for the case where the thickness of the Al_2_O_3_ is kept constant at 80 nm and the thicknesses of the MoO_3_ layer are 40 nm, 60 nm, 80 nm, and 100 nm. The intensity of reflectance decreases by about 7%, while the transmittance decreases significantly by 20% when the thickness of the MoO_3_ layer is increased from 40 nm to 100 nm. The reference (bare) glass has reflectance and transmittance of about 5% and 96% across the visible range. Generally, it is noticed that only varying shades of gray are produced by the Type A multi-layer. The simulated color profile for Type A multi-layer is shown in Figure 4c. Generally, it is shown that it is difficult to achieve bright colors (other than grey) to meet aesthetic considerations, even when thick layers of MoO_3_ and Al_2_O_3_ are deposited. In cases where thick layers are deposited, the overall fabrication becomes expensive and requires long processing time. Therefore, from the perspective of large-scale commercial optical applications, the Type A multi-layer is not suitable.

**Figure 5:**
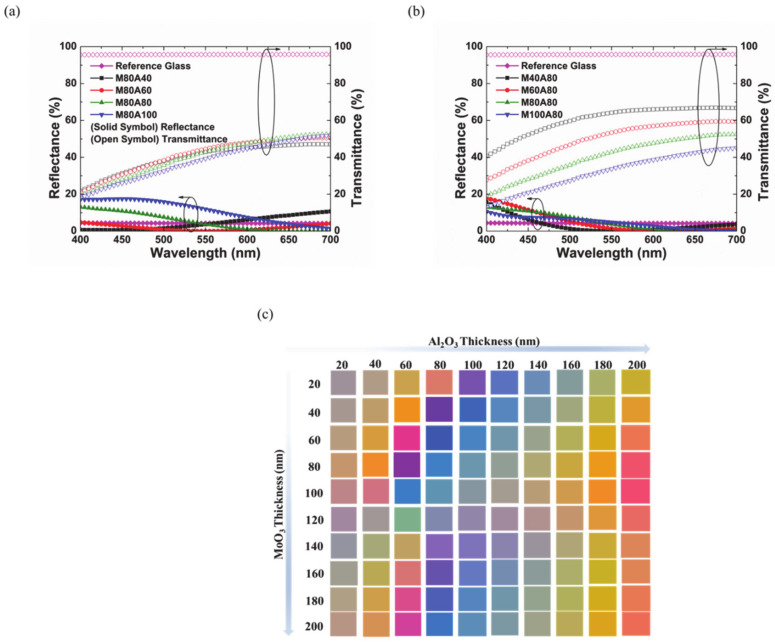
Reflectance and transmittance spectra of the Type B multi-layer (**a**) with 80 nm of MoO_3_ and 40 nm, 60 nm, 80 nm, and 100 nm of Al_2_O_3_ and (**b**) with 80 nm of Al_2_O_3_ and 40 nm, 60 nm, 80 nm, and 100 nm of MoO_3_. (**c**) Simulated color profile of the Type B multi-layer.

The reflectance and transmittance spectra of the Type B multi-layer with 80 nm of MoO_3_ and 40 nm, 60 nm, 80 nm, and 100 nm of Al_2_O_3_ are shown in Figure 5a. It is shown that, for a beam within wavelengths of 400 nm and 460 nm, maximum reflectance is increased by about 20%, while the transmittance is decreased marginally by 5% when the thickness of the Al_2_O_3_ layer is increased from 40 nm to 100 nm. This can be attributed to the increased constructive interference for the reflected beams within 400 nm and 460 nm. The glasses with 60 nm, 80 nm, and 100 nm of an Al_2_O_3_ layer showed similar shades of color. However, the film with a 40 nm thick Al_2_O_3_ layer showed a different color spectrum with increasing reflectance and decreasing transmittance for beams within the wavelength of 500 nm and 700 nm. Thus, it demonstrates that increasing the thickness of Al_2_O_3_ beyond 40 nm does not significantly change or tune the color from the reflected beam.

Next, we verified the effect of thickness of the MoO_3_ on the thin film interference of the Type B multi-layer. Figure 5b shows the reflectance and transmittance spectra of a Type B multi-layer with 80 nm of an Al_2_O_3_ layer and 40 nm, 60 nm, 80 nm, and 100 nm of a MoO_3_ layer. It can be noticed that a different color spectrum is obtained for each film with 40 nm, 60 nm, 80 nm, and 100 nm of MoO_3_. This can be attributed to different OPDs obtained for the Type B multi-layer when the thickness of the MoO_3_ (underlying layer with higher refractive index) is changed. For each incident beam, a unique constructive interference for different film thicknesses is achieved. It is also shown that increasing the thickness of the MoO_3_ layer increases the constructive interference of the transmitted beams, thus causing a reduction in the transmittance of the film. It is most preferrable to have freedom of color choice for commercial application in BIPV. Figure 5c shows the simulated color profile for the Type B multi-layer. It is demonstrated that bright colors can be achieved even with MoO_3_ and Al_2_O_3_ layer thicknesses below 100 nm at a low cost and within a short processing time. Thus, our fabricated color films are based on the Type B multi-layer. Colored glasses of choice can be easily fabricated by changing the thickness of the MoO_3_ layer.

### 3.3. Optical Characteristics of Samples

**Figure 6:**
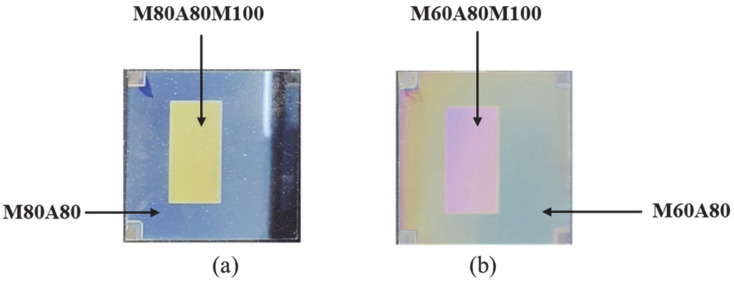
Photograph of (**a**) M80A80 and M80A80M100, (**b**) M60A80 and M60A80M100 colored glasses.

The MoO_3_ and Al_2_O_3_ films were deposited by means of a thermal evaporator and radio frequency reactive sputtering, respectively. Four colored glass derivatives of the Type B multi-layer, herein referred to as M80A80, M80A80M100, M60A80, and M60A80M100, were fabricated based on glass/MoO_3_(80 nm)/Al_2_O_3_(80 nm), glass/MoO_3_(80 nm)/Al_2_O_3_(80 nm)/MoO_3_(100 nm), glass/MoO_3_(60 nm)/Al_2_O_3_(80 nm), and glass/MoO_3_(60 nm)/Al_2_O_3_(80 nm)/MoO_3_(100 nm) multi-layers, respectively. The actual photographs of the fabricated M80A80, M80A80M100, M60A80, and M60A80M100 colored glasses are shown in Figure 6. M80A80M100 and M60A80M100 were achieved by depositing MoO_3_ (100 nm) layers on M80A80 and M60A80, respectively, via a shadow mask. M80A80 and M80A80M100 show shades of blue and yellow colored glasses, as shown in Figure 6a. Figure 6b shows the M60A80 and M60A80M100 samples exhibiting green and pink colors, respectively. Generally, the shades of blue colors achieved in the M80A80 and M60A80 samples are comparable to that obtained from the simulation, as shown in Figure 5c. Notable color deviations, especially as seen in the M60A80 sample, may be due to a variation in the thickness of deposited layers from those in the simulation.

**Figure 7:**
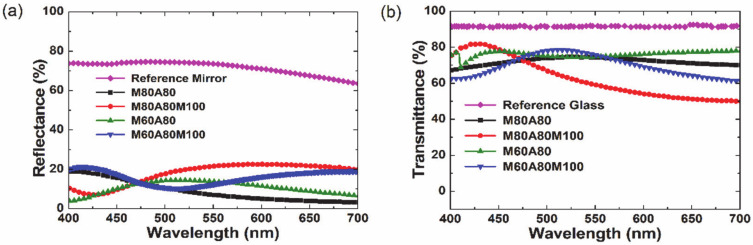
(**a**) Reflectance and (**b**) transmittance spectra of M80A80, M80A80M100, M60A80, and M60A80M100 colored glasses.

Figure 7a shows the absolute reflectance of the reference mirror and the relative reflectance spectra of M80A80, M80A80M100, M60A80, and M60A80M100 colored glasses within the visible range. The reference mirror shows a maximum reflectance of 75% at 485 nm and roll off steadily to 63% at 700 nm. The M80A80 and M60A80 color samples achieve maximum reflectance of 19% at 400 nm and of 15% at 514 nm, respectively. This is in good agreement with the simulation results in Figure 5b that changing the thickness of the MoO_3_ layer changes the color spectrum. M80A80M100 and M60A80M100 colored glasses showed brighter colors with higher reflectance than the M80A80 and M60A80 samples. This can be attributed to enhanced constructive interference caused by the additional MoO_3_ (100 nm) layer in the M80A80M100 and M60A80M100 samples. The colors from the M80A80 and M60A80 are the reflected beams from the Air/MoO_3_, Al_2_O_3_/MoO_3_, and MoO_3_/glass interfaces, respectively. On the other hand, cumulated reflected beams from the Air/MoO_3_, MoO_3_/Al_2_O_3_, Al_2_O_3_/MoO_3_^,^ and MoO_3_/glass interfaces resulted in bright colors (yellow and pink) with broad reflection spectra in the M80A80M100 and M60A80M100 samples. Thus, for the broad spectrum of incident light, total reflectance is enhanced in the multi-layer composed of three layers as compared to those composed of two layers. The maximum reflectance of M80A80M100 and M60A80M100 colored glasses are 23% at 590 nm and 21% at 411 nm, respectively. The above findings demonstrate that blue and green are primarily achievable from MoO_3_ and Al_2_O_3_ bilayers while brighter colors such as yellow and pink can be secondarily obtained from the three-layered structures of MoO_3_ and Al_2_O_3_.

The transmittance spectra of the reference glass, M80A80, M80A80M100, M60A80, and M60A80M100 colored glasses within the visible range are shown in Figure 7b. The transmittance of the reference glass is about 91% across the visible range. Maximum transmittance of 82% at 428 nm was achieved for M80A80M100 colored glass, followed by M60A80M100 colored glass with maximum transmittance of 79% at 511 nm. The M80A80 and M60A80 colored glasses show a maximum transmittance of 75%. These results indicate that strong constructive interference of reflected and transmitted beams is achieved by the three-layered derivatives of multi-layer Type B. The reflectance and transmittance of the colored glasses can be tuned by the high refractive index material: MoO_3_. It can be observed that the spectra of the samples (M80A80 and M60A80) exhibit some deviation from the simulation spectra. This could be attributed to the difference in the actual thickness deposited from that used in the simulation. Moreover, the optical interference (scattering, reflectance, etc.) at the substrate could be different in both cases. Nonetheless, the main thrust of the simulation to determine a suitable multi-layer structure (Type B) was demonstrated in this investigation. Subsequent investigations will optimize the simulations further, so that there is a better agreement with the experiment.

In BIPV systems, it is desired that front colored glasses have high transmittance to ensure high efficiency. We successfully demonstrated that we can achieve colored glasses with high transmittance of more than 82%. Generally, most colored glasses cause minimal loss of efficiency due to the reflectance by the color filters [33]. It is investigated that the efficiency of mono-crystalline silicon-framed standard-size PV modules with Kromatix panels—black, green, blue, grey, and gold panels--integrated in a roof caused minimal loss of efficiency [33]. The difference in efficiency between black (100%) and colored modules can be noted as following: green (91%), blue (87%), grey (83%), and gold (83%) [33]. The efficiency of the PV modules with the colored glasses depends on the color and the transmittance of each glass. Based on these results, it can be projected that our M80A80M100 colored glasses with a maximum transmittance of 82% could cause a less than 20% decrease in efficiency.

**Figure 8:**
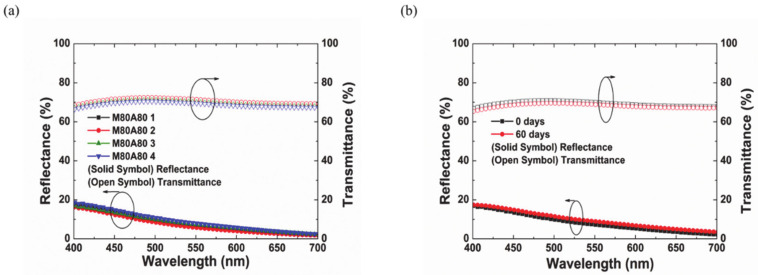
Reflectance spectra of colored glasses for (**a**) uniformity and (**b**) stability analysis.

The optical characteristics of four M80A80 samples made by our deposition techniques were further checked for uniformity. Figure 8a shows the reflectance and the transmittance spectra of the four samples. The samples were positioned on the substrate holder within a circular radius of 10 cm. The samples showed similar reflectance and transmittance spectra, demonstrating that a large and uniform color films for commercial application can be produced by our fabrication method.

When building exterior walls with BIPV systems, the stability of the color film against environmental conditions must be ensured. Figure 8b shows the reflectance and the transmittance of M80A80 colored glass maintained in ambient condition over two months. It was confirmed that the degree of change in the multi-layer film and its optical characteristics over the period is insignificant. This demonstrates the stability of the color films made by the multi-layer metal oxides.

## 4. Conclusions

We investigated MoO_3_ and Al_2_O_3_ multi-layers as color coatings for colored glasses of BIPV systems and developed a process technology to realize it. It was investigated in a simulation based on wave optics that glass/MoO_3_/Al_2_O_3_ multi-layer type offers better color design freedom and a cheaper fabrication process when compared to the glass/Al_2_O_3_/MoO_3_ multi-layer type. We demonstrated that different wavelengths of light can create constructive interference for different film thicknesses if the incident beam travels from a layer with lower refractive index into a layer with higher refractive index. The large difference in refractive index of the glass/MoO_3_/Al_2_O_3_ multi-layer ensured that various colored glasses can be achieved from thin film depositions. Blue and green colored glasses were achieved by MoO_3_ (80 nm)/Al_2_O_3_ (80 nm) and MoO_3_ (60 nm)/Al_2_O_3_ (80 nm) bilayer depositions, respectively, while yellow and pink colored glasses were obtained from MoO_3_ (80 nm)/Al_2_O_3_ (80 nm)/MoO_3_ (100 nm) and MoO_3_ (60 nm)/Al_2_O_3_ (80 nm)/MoO_3_ (100 nm) depositions, respectively. A high transmittance of 82% at 428 nm and reflectance of 23% at 590 nm were obtained for the MoO_3_ (80 nm)/Al_2_O_3_ (80 nm)/MoO_3_ (100 nm) film. Moreover, stable, large, and uniform colored glasses are made possible by our fabrication method. The glass/MoO_3_/Al_2_O_3_ multi-layer structure and fabrication technique proposed allows for cheap and desirable colored glasses to be implemented in commercial BIPV systems, and other photonic devices.

## Data Availability

The data presented in this study are available on request from the corresponding author.
